# Time trends in the regional distribution of physicians, nurses and midwives in Europe

**DOI:** 10.1186/s12913-020-05760-y

**Published:** 2020-10-12

**Authors:** Juliane Winkelmann, Ulrike Muench, Claudia B. Maier

**Affiliations:** 1 Department of Healthcare Management, Technische Universität Berlin, H 80, Straße des 17. Juni 135, 10623 Berlin, Germany; 2 Department of Social and Behavioural Sciences, University of California San Francisco, School of Nursing, 3333 California Street, Ste 455, San Francisco, CA 94118 USA; 3 Philip R. Lee Institute for Health Policy Studies, University of California San Francisco, School of Medicine, 3333 California Street, Ste 455, San Francisco, CA 94118 USA; 4 Center for Health Outcomes and Policy Research, University of Pennsylvania, School of Nursing, Claire Fagin Hall, 418 Curie Blvd, Philadelphia, PA 19104 USA

**Keywords:** Health workforce research, Health workforce policy, Health professionals, Europe, Health workforce distribution, Supply, Shortage, Rural, Urban

## Abstract

**Background:**

Country-level data suggest large differences in the supply of health professionals among European countries. However, little is know about the regional supply of health professionals taking a cross-country comparative perspective. The aim of the study was to analyse the regional distribution of physicians, nurses and midwives in the highest and lowest density regions in Europe and examine time trends.

**Methods:**

We used Eurostat data and descriptive statistics to assess the density of physicians, nurses and midwives at national and regional levels (Nomenclature of Territorial Units for Statistics (NUTS) 2 regions) for 2017 and time trends (2005–2017). To ensure cross-country comparability we applied a set of criteria (working status, availability over time, geographic availability, source). This resulted in 14 European Union (EU) countries and Switzerland being available for the physician analysis and eight countries for the nurses and midwives analysis. Density rates per population were analysed at national and NUTS 2 level, of which regions with the highest and lowest density of physicians, nurses and midwives were identified. We examined changes over time in regional distributions, using percentage change and Compound Annual Growth Rate (CAGR).

**Results:**

There was a 2.4-fold difference in the physician density between the highest and lowest density countries (Austria national average: 513, Poland 241.6 per 100,000) and a 3.5-fold difference among nurses (Denmark: 1702.5, Bulgaria: 483.0). Differences by regions across Europe were higher than cross-country variations and varied up to 5.5-fold for physicians and 4.4-fold for nurses/midwives and did not improve over time. Capitals and/or major cities in all countries showed a markedly higher supply of physicians than more sparsely populated regions while the density of nurses and midwives tended to be higher in more sparsely populated areas. Over time, physician rates increased faster than density levels of nurses and midwives.

**Conclusions:**

The study shows for the first time the large variation in health workforce supply at regional levels and time trends by professions across the European region. This highlights the importance for countries to routinely collect data in sub-national geographic areas to develop integrated health workforce policies for health professionals at regional levels.

## Background

Health professionals are the backbone of all health systems [1]. Evidence suggests that the supply of health professionals and their geographical distribution is associated with population health outcomes, such as maternal mortality, avoidable cancer mortality and lower hospitalization rates for ambulatory care sensitive conditions [2, 3]. Ensuring a sufficient and balanced supply of a well-educated health workforce is therefore of high importance to many countries, especially in light of increasing chronic conditions, ageing populations, epidemiological and socio-cultural changes [4–8]. While the demand for health care and health professionals is expected to grow, the health workforce itself is ageing. In many countries, fewer individuals are choosing to become physicians or nurses, often as a result of high workload, shift and weekend work, stress and low remuneration. This leaves positions unfilled, leading to shortages for certain professions, specialties and geographic areas [9].

Data on the supply of health professionals show large variations worldwide, with low income countries facing very low density levels compared to high income countries [5]. To date, the majority of worforce supply data exist at the national level, allowing for cross-country comparisons [10]. Yet, many European countries face regional workforce imbalances. While urban areas attract larger numbers of physicians and other health professionals [11, 12] rural and remote regions face an undersupply of physicians [13, 14]. The goal of this paper is to better understand the variation in workforce supply at regional level. Regional imbalances of physician densities affect many health care services, and access to primary care services in particular. In France, for example, about 23% of the mainland population report difficulties in finding a GP closer than a thirty-minute drive from their home [12, 15]. The shortage of physicians is not only a problem in rural areas but also in urban disadvantaged areas that experience higher rates of poverty, unemployment and people on welfare. The situation is expected to deteriorate with the increasing average age of physicians and the difficulties of replacing them in these areas [16].

Various countries are introducing policies and programmes to attract and retain health professionals in rural and underserved areas [4, 14, 17, 18]. Frequent measures include financial incentives for physicians to move and establish a practice in rural areas (ibid.). Others are non-financial in nature, including improved work-life balance, additional training opportunities and professional support through telemedicine, establishing educational institutions in rural areas, and admitting health professional students from rural backgrounds since these individuals are more likely to practice in rural areas following graduation [18, 19]. However, the success of these interventions remains unclear, largely due to a lack of data availability [14], and the majority of policies that targets the physician workforce.

Prior research has paid little attention to the distributions of health professionals across countries by region [10]. This study aims to: first, analyse the density of physicians, nurses and midwives at national and regional levels in a selection of European Union (EU) countries and European Free Trade Association (EFTA) countries, belonging to the EU’s single market with comparable data. Second, to identify the regions with the highest and lowest density, and third, to assess changes in health professional densities over time.

## Methods

The study used routinely collected data from EUROSTAT on the following health professions: physicians; nurses and midwives. These professional groups make up the majority of the health workforce [20] and have been subject of strategies to alleviate health workforce shortages in many countries. In the EU, these professions are regulated under the EU’s directives on the mutual recognition of diploma and therefore, show minimum levels of harmonisation [21, 22]. EUROSTAT data include the total number of health care professionals and density per 100,000 population. The time trend analysis was based on over 10 years of data spanning the years 2005 to 2017, or nearest available year.

### Availability of national and regional level data.

Data from EUROSTAT are available on the EU, its Member States, EFTA countries, as well as EU candidate countries and potential candidates at national (total average) and regional levels. National data are collected through the Joint Questionnaire on Non-Monetary Health Care Statistics and regional data (at NUTS 2 level) are collected through EUROSTAT. The latter are based on administrative sources, relying on professionals’ registries, labour force surveys or sample surveys [23]. To obtain and compare regional statistics, the EU uses a standardised classification system, called the Nomenclature of Territorial Units for Statistics (NUTS). The NUTS system comprises three categories to capture geographical variation: NUTS 1 regions are larger socio-economic regions (with 104 regions in Europe as of 2018, between 3 and 7 million inhabitants), NUTS 2 are medium-level regions (281 regions, between 800,000 and 3 million inhabitants) and NUTS 3 make up the smallest regional unit (1348 regions, between 150,000 and 800,000 inhabitants) [24]. In most countries, each NUTS level corresponds to a certain administrative level or an aggregation of administrative units [25].

The regions differ with respect to land area, population, economic strength and administrative importance. Moreover, given the different aggregation of administrative units (also due to population and size of territory), the number of NUTS 2 regions per country differs. Slovenia and Croatia for example only have two NUTS 2 regions. Various small countries but also the UK only have one NUTS 2 region or only data at national level [23].

### Outcome measure

To assess density of the physician, nurse and midwife workforce at national and regional levels, we followed the International Standard Classification of Occupations (ISCO) used by EUROSTAT. There are three underlying concepts with regard to definitions of employment of healthcare personnel: ‘practising’, ‘professionally active’ and ‘licensed’. Only practicing physicians, nurses and midwives were included as the concept of ‘practising’ best describes the availability of health professionals providing direct patient care.

Data on physicians included those who have completed studies in medicine at university level and who were licensed to practice [26]. Data on nurses and midwives (ISCO codes 2222, 3222, 2221 and 3221) included general, clinical, district and specialist nurses, nurse anaesthetists, nurse educators, nurse practitioners, public health nurses. The professions are regulated by the European Commission and comprise therefore minimum levels of education [27]. EUROSTAT combines data on nurses and midwives as standard practice at NUTS 2 level.

### Country selection

We included countries that belonged to the EU’s single market, had data available at NUTS 2 level or smaller (at least two NUTS 2 regions per country), and had collected data over the study period or at minimum of six consecutive years between 2005 and 2017. To improve cross-country comparability, we limited the analysis to countries that used the EUROSTAT indicator of “practicing” physicians, nurses and midwives (professionally active or licensed were excluded) and used consistent data sources over time, obtained via registers, surveys or reports by authoritative sources [28, 29].

Following these inclusion criteria, 15 countries were included in the analysis of physician densities (Austria, Belgium, Bulgaria, Croatia, Czech Republic, Denmark, Germany, Hungary, Netherlands, Poland, Portugal, Romania, Slovak Republic, Slovenia, Sweden and Switzerland) and eight countries were included in the analysis of nurses and midwives (Bulgaria, Czech Republic, Croatia, Denmark, Hungary, Poland, Slovenia and Sweden). Overall, data on physicians were available for most years while data on nurses/midwives were generally available up to the year 2015 and less complete at regional level. Availability of data, definitions used for physicians and nurses/midwives and reasons for exclusion are specified in Table 1.

**Table 1:** Data availability, source and definition of outcome measure based on EUROSTAT

Country	Geographic availability	Availability over time	Source	Working status (P/LP/PA) and other specific information	Included	Exclusion reason
**Austria**						
*Physicians*	national, regional	2005–2017	Registry (head count)	P	X	
*Nurses/midwives*	national, regional	2005–2015	Hospital statistics	working in hospitals only	–	Only hospital nurses
**Belgium**						
*Physicians*	national, regional	2011–2017	Annual Report, Head count data	P, excludes physicians in training, includes stomatologists, only accounts for physicians with minimum volume of patient contact^1^	X	
*Nurses/midwives*	[Data under revision]				–	No data available
**Bulgaria**						
*Physicians*	national, regional	2005–2017	Exhaustive annual survey (head count)	P, only with labour contract	X	
*Nurses/midwives*	national, regional	2005–2015	Exhaustive annual survey (head count)	P, only with NHS contract	X	
**Czech Republic**						
*Physicians*	national, regional	2005–2013	Registry	P, only employees on payroll included	X	
*Nurses/midwives*	national, regional	2005–2015	Annual report (Institute of Health Information and Statistics)	P, double counting for those working in two institutions.	X	
**Croatia**						
*Physicians*	national, regional	2010–2017	Registry	P, maxillofacial surgeons included (until 2008)	X	
*Nurses/midwives*	national, regional	2010–2015	Registry	P	X	
**Denmark**						
*Physicians*	national, regional	2007–2016	Registry	P	X	
*Nurses/midwives*	national, regional	2007–2014	Registry	P	X	
**Finland**						
*Physicians*	national, regional	2005–2014 (national), 2011–2014 (3 of 5 regions)	Registry	LP (not retired), since 2009 estimations based on PA	–	Incomplete times series
*Nurses/midwives*	national, regional	2010–2011	Registry	Includes nurses active in health care	–	Incomplete times series
**France**						
*Physicians*	national, regional^1^	2005–2017	Registry	P since 2011 and interns and residents are excluded; PA at regional level	–	Deviating definition
*Nurses/midwives*	national, regional	2005–2016	Registry	PA	–	Deviating definition
**Germany**						
*Physicians*	national, regional	2005–2017	Registry	P	X	
*Nurses/midwives*	national				–	No regional data
**Greece**						
*Physicians*	national, regional	2005–2017	Survey	LP	–	Deviating definition
*Nurses/midwives*	national, regional	2005–2015	Census	Only working in hospitals.	–	Only hospital nurses
**Hungary**						
*Physicians*	national, regional	2005–2016	Registry (Head count)	P	X	
*Nurses/midwives*	national, regional	2006–2015	Report personnel of health service	P	X	
**Italy**						
*Physicians*	national, regional	2005–2017	Total survey & estimations	Different definitions used	–	Numbers obtained via survey and estimations Numbers obtained via survey and estimations
*Nurses/midwives*	national, regional	2008–2016	Estimations based on registry (national); 2008–2010 Registry, 2011–2012 LFS, 2013 onwards estimations based on registry (regional)		–	
**Netherlands**						
*Physicians*	national, regional	2005–2017	Registry	P	X	
*Nurses/midwives*	national, regional	2005–2013	Until 2008 estimates, Registry and database (from 2014 onwards)	P (since 2014, but no data available)	–	Numbers obtained via estimations (until 2008) and deviating definition
**Norway**						
*Physicians*	national, regional	2005–2017	Registry	Data from 2009 to 2012 covers physicians, nurses and midwives also within HP4 (professionals in ancillary services, i.e. mostly without direct patient contact)	–	Deviating definition
*Nurses/midwives*	national, regional	2005–2015	Registry		–	Deviating definition
**Poland**						
*Physicians*	national, regional	2005–2016	Ministries and Central Statistical Office	Physicians working in prisons excluded	X	
*Nurses/midwives*		2005–2015	Ministries and Central Statistical Office, based on headcounts	Nurses working in prisons excluded	X	
**Portugal**						
*Physicians*	national, regional	2005–2017	Registry (head count)	LP	–	Deviating definition
*Nurses/midwives*	national, regional	2005–2016	Registry	PA	–	Deviating definition
**Romania**						
*Physicians*	national, regional	2005–2017^2^	Survey	P (as of 2008), includes oral and maxillofacial surgeons	X	
*Nurses/midwives*		2005–2015	Survey	Not only practicing midwives (2009–2014), data on nurses refers to ancillary medical staff (2000–2009)	–	Deviating definition
**Slovakia**						
*Physicians*	national, regional	2005–2017	Registry (national), Registry and Report (regional)	P	X	
*Nurses/midwives*	national, regional	2009–2014	Annual report	PA	–	Deviating definition
**Slovenia**						
*Physicians*	national, regional	2005–2017	Registry	P	X	
*Nurses/midwives*	national, regional	2005–2015 (no data for 2014)	Registry	P	X	
**Spain**						
*Physicians*	national, regional	2005–2017	LFS (times series data not fully comparable)	Odontologists and dentists included (regional level, until 2010/2011)	–	Comparability over time restricted
*Nurses/midwives*	national, regional	2005–2015	LFS (times series data not fully comparable)		–	Comparability over time restricted
**Sweden**						
*Physicians*	national, regional	2005–2016	Registry (head count)	P	X	
*Nurses/midwives*	national, regional	2005–2014	Registry	P	X	
**Switzerland**						
*Physicians*	national, regional	2007–2017	Yearly census	P	X	
*Nurses/midwives*	national	2005–2015	Federal statistical office, Estimates until 2009	Midwives in ambulatory sector excluded	–	Deviating definition

*Notes*: *LFS* Labour Force Survey, *P* Practicing physicians, nurses, midwives, *LP* (physicians, nurses, midwives) licensed to practice, *NHS* National Health System, *PA* Professionally active physicians, nurses, midwives; *HP* OECD/EUROSTAT classification of Health Care Providers (HP1: hospitals, HP2:residential long-term care facilities, HP3: ambulatory care providers, HP4: ancillary care providers). ^1^ Data on health professionals in the five French overseas departments is only available for 2015–2016. ^1^ In Belgium, a minimum threshold of activities (500 consultations per year) is set for general practitioners to be considered to be practising, resulting in an under-estimation compared with other countries which do not set such a threshold (OECD Health at a Glance Europe 2018); ^2^ until 2007 physicians working in administration, research and in other posts that exclude direct contact with patients could not be totally excluded

Sources: [30] [26, 31];

### Data analysis

We retrieved the number of physicians and nurses and midwives per 100,000 population from the EUROSTAT database for the year 2017 or nearest available. The rate of health professionals per population was used to compare densities of professionals across countries, comparing density levels at national and NUTS 2 level. It is the most commonly used measure to assess the density and availability of a country’s health workforce [32]. In addition, we compared health professional rates to general population density to capture the degree of urbanisation of the regions. In order to analyse changes over time in the national and regional distributions of physicians and nurses and midwives, we used data between the years 2005 to 2017 or nearest available year. We calculated the yearly percentage change beginning with 2005 to 2017 (or year available) by country, region and profession type. Moreover, we calculated the compound annual growth rate (CAGR) to assess the relative growth of the professions. The CAGR is a common method in health services research and other disciplines to analyse time trends. Compared to calculating yearly percentage change, CAGR has the advantage that it smoothes differences from year to year [33, 34].

## Results

### Density of physicians

**Fig. 1:**
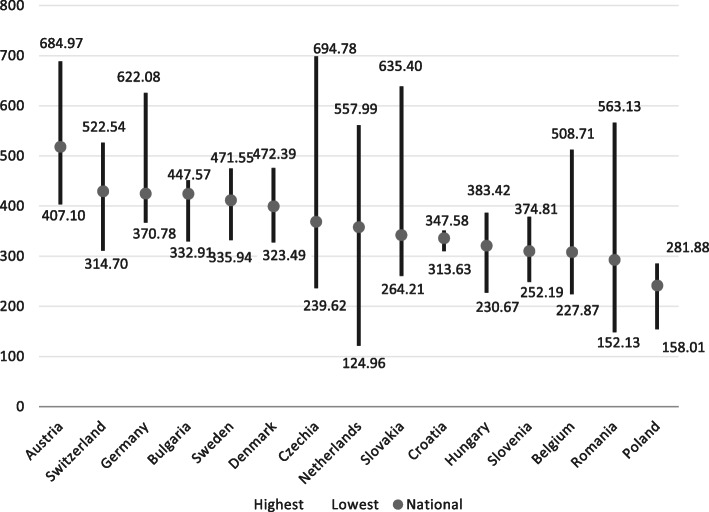
Physician distribution at national level and by region (NUTS 2) per 100,000 population in 15 countries in 2017 (or latest available year). Notes: Data end points refer to the region (NUTS2) with the highest and lowest density of physicians, and national average across all NUTS2 regions. Year: 2017, except for: Czech Republic (2013), Denmark (2016), Hungary (2016), Poland (2016), Sweden (2016). ^1^ In Belgium, a minimum threshold of activities (500 consultations per year) is set for general practitioners to be considered to be practising, resulting in an under-estimation compared with other countries which do not set such a threshold (OECD 2018, Health at Glance 2018). Regions with highest/lowest physician densities by country: Austria (Vienna/Burgenland), Switzerland (Zurich/Central Switzerland), Germany (Hambourg/Brandenburg), Bulgaria (North West/North Central), Sweden (Stockholm/North-Central Sweden), Denmark (Capital/North Jutland), Czechia (Praha/Central Bohemia), Netherlands (Utrecht/Flevoland), Slovakia (Bratislava/West Slovakia), Croatia (Continental Croatia/Adriatic Croatia), Hungary (Central Hungary/North Hungary), Slovenia (West Slovenia/East Slovenia), Belgium (Brabant Wallon/Luxembourg), Romania (Bucharest-Ilfov/South-Muntenia), Poland (Łódzkie/Wielkopolskie province). Sources: EUROSTAT data on physicians by NUTS2 region

The national density of practicing physicians varied across the 15 countries, with a 2.4-fold difference between the country with the highest (Austria, national average 518.3 per 100,000 population in 2017) and lowest physician density (Poland, 241.6, per 100,000) (Fig. 1). The average national physician density across 14 countries (excluding Czech Republic as data were only available up to 2013) in 2016 was 361.6 physicians per 100,000 population which is close to the EU28 average of 360.1 in the same year [15, 35].

Comparing physician densities at regional NUTS 2 level showed large imbalances in the distribution of physicians across countries. Rates varied widely across regions, ranging from 125 in the Netherlands (Flevoland) and 152.1 physicians in Romania (South-Muntenia) to 694.8 physicians in the metropolitan region of Praha (Czech Republic, data refer to 2013) and 685 physicians per 100,000 population in the Vienna region (Austria) (Fig. 1). Overall, there was a 5.5-fold difference between the two regions with the highest and lowest density, more than twice as high compared to national density levels.

Within countries, regional imbalances also existed. The Netherlands and the Czech Republic had the highest within-country differences in terms of physician density (4.5-fold and 2.9-fold, respectively). The lowest geographical imbalances were found for Croatia and Bulgaria. In all countries, except Bulgaria, the highest rates of physicians per population were identified in areas with highest population densities, where capitals or large cities are located. The capital regions of Praha (694.8), Vienna (685) and Bratislava (635.4) showed the highest regional density levels of physicians, followed by Hambourg (622.1 physicians per 100,000 inhabitants (Table 2). By contrast, physician density was lowest in sparsely populated regions in Belgium, Croatia, Denmark, Slovenia or less densely populated areas in Bulgaria, Czech Republic, Germany, Hungary, Netherlands, Austria, Poland, Sweden, Switzerland. However, in three countries the most sparsely populated areas had among the highest national physician rates per capita: Bulgaria (North West, 447.6 physicians), Hungary (Southern Transdanubia 328.2 physicians) and Poland (Podlaskie 274.5 physicians per 100,000 population).

**Table 2:** Geographical supply of physicians (practicing) between highest and lowest density regions and national average, 2005–2017 (or nearest) (per 100,000 population)

Country average, *highest/lowest region*	2005	2006	2007	2008	2009	2010	2011	2012	2013	2014	2015	2016	2017	PD (per sq. km), 2017 or nearest	PC change(2005–2017 or nearest)	CAGR
Austria	431,68	444,96	453,78	460,41	468,91	479,53	484,22	489,54	498,85	504,61	509,12	512,96	518,28	106,80	20,06	1,42
*Vienna*	625,26	640,92	644,69	646,07	651,73	665,05	663,64	664,19	685,29	688,54	683,97	681,16	684,97	4.754,9	9,55	0,70
*Burgenland*	305,84	311,52	321,19	328,51	339,64	352,29	364,33	374,52	377,98	377,58	387,32	397,97	407,10	79,70	33,11	2,22
Belgium	287,07	288,59	290,50	292,07	292,48	291,34	291,55	293,36	295,71	297,55	301,75	307,41	308,29	373,60	7,39	0,55
*Brabant Wallon*							472,52	480,68	486,61	487,05	496,66	499,32	508,71	365,20	7,66	1,06
*Luxembourg*							219,70	221,54	226,66	224,18	224,31	228,92	227,87	63,80	3,72	0,52
Bulgaria	367,86	369,50	369,53	366,42	375,47	378,10	386,26	391,45	397,67	398,69	404,54	413,76	424,49	64,30	15,39	1,11
*North West*	344,49	359,90	368,44	373,05	390,98	403,60	409,30	408,18	416,43	418,53	422,88	431,15	447,57	40,50	29,92	2,03
*North Central*	291,77	290,68	291,08	297,32	302,62	309,58	307,88	316,26	314,34	312,52	317,98	323,77	332,91	54,6	14,10	1,02
Croatia	257,44	260,84	273,58	273,75	274,32	286,38	291,62	299,18	303,35	314,02	319,15	323,65	336,21	73,9	30,57	2,07
*Cont. Croatia*						293,13	298,06	307,95	312,05	320,76	327,15	333,73	347,58	87,50	14,67	1,73
*Adriatic Croatia*						272,64	278,54	281,37	285,74	300,41	303,09	303,49	313,63	56,40	23,29	2,65
Czechia	356,28	357,41	357,47	355,54	357,63	359,55	363,67	367,47	368,79					137,30	3,51	0,38
Praha	675,10	685,80	685,64	671,42	674,38	666,38	672,54	678,90	694,78					2.654,70	2,92	0,32
*Central Bohemia*	263,45	259,60	253,23	249,76	243,53	242,25	240,46	243,16	239,62					125,60	−9,50	−1,05
Denmark	333,47	342,36	345,81	357,94	365,12	372,59	378,92	383,76	384,94	387,74	392,93	399,82		137,30	19,90	1,52
*Capital*			438,62	442,55	448,40	450,99	448,98	453,25	456,07	458,39	463,27	472,39		745,40	7,70	0,74
*North Jutland*			282,40	285,50	297,03	298,42	323,32	323,89	319,81	325,86	321,54	323,49		76,20	14,55	1,37
Germany	339,53	343,67	348,63	354,06	361,89	374,81	387,70	394,64	403,50	410,82	413,93	418,65	424,88	234,00	25,44	1,74
*Hambourg*	468,94	472,70	483,32	498,66	521,72	549,61	577,82	588,10	597,54	604,25	611,85	614,42	622,08	2.564,10	32,66	2,20
*Brandenburg*	288,62	292,76	296,65	303,03	309,45	318,62	3 27,69	336,22	343,48	351,45	358,18	363,81	370,78	86,10	28,47	1,95
Hungary	278,13*	303,58	280,33*	309,06*	302,08	286,86*	296,22	308,87	320,91	332,35	309,72^a^	321,12	332,48	107,30	19,54	1,38
*Central Hungary*	406,84	436,78	415,36	447,96	432,98	382,12	390,22	407,21	426,77	414,21	396,94	383,42		441,30	−5,76	−0,49
*North Hungary*	191,19	205,82	182,23	209,03	204,74	185,95	211,51	214,29	228,62	240,25	211,94	230,67	226,96	85,70	18,71	1,33
Netherlands	270,75	279,50	279,35	287,02	292,26	296,36	313,27	325,34	331,35	342,50	348,72	353,68	358,22	501,10	32,31	2,18
*Utrecht*	405,81	422,75	424,17	437,02	451,52	453,12	480,80	502,25	509,49	528,44	537,29	545,87	557,99	918,10	37,50	2,48
*Flevoland*	134,96	132,87	126,94	127,27	126,28	129,77	134,60	135,02	132,28	131,47	128,81	123,66	124,96	285,50	−7,41	−0,59
Poland	213,81*	217,92	219,09	216,12	217,47	218,70	221,27	223,38	224,09	230,68	232,81	241,58		123,60	12,99	1,02
*Łódzkie*	251,76*	252,66	244,86	249,34	250,00	245,79	249,48	254,40	253,17	276,42	268,70	281,88		136,70	11,96	0,95
*Wielkopolskie*	176,41*	173,28	170,37	168,41	151,84	151,37	145,01	145,80	147,05	159,76	151,65	158,01		117,10	−10,43	−0,91
Romania				221,33	225,71	236,86	246,46	261,05	264,36	269,83	276,58	284,10	292,68	83,60	32,24	2,83
*Bucharest-Ilfov*				483,77	480,95	516,70	498,75	514,05	509,46	529,34	533,72	553,11	563,13	1.308,00	16,40	1,53
*South-Muntenia*				127,09	130,96	136,97	137,16	142,06	147,32	148,09	149,62	152,08	152,13	88,10	19,70	1,81
Slovenia	234,54	236,19	239,46	240,14	240,97	243,05	249,46	254,14	262,92	277,02	282,53	301,40	310,11	102,60	32,22	2,17
*West Slovenia*				307,26	304,27	301,32	306,92	302,20	306,85	324,74	325,86	354,95	374,81	124,90	21,98	2,01
*East Slovenia*				183,11	186,65	192,70	199,57	212,17	224,28	234,80	244,04	253,65	252,19	88,40	37,73	3,25
Slovakia	303,71	317,14	338,98	336,87	330,42	335,90	330,64	336,44	339,07	342,78	345,13	347,35	342,11	111,70	12,64	0,92
*Bratislava*	551,20	618,05	698,80	677,39	680,49	668,82	668,56	681,26	686,72	678,22	666,37	655,75	635,40	319,80	15,28	1,10
*West Slovakia*	243,16	248,25	262,20	260,14	249,49	254,35	251,17	257,52	258,95	263,27	263,27	263,56	264,21	123,3	8,66	0,64
Sweden	351,42	360,22	368,20	374,21	381,24	388,29	396,42	404,67	413,35	420,20	427,06	411,68^a^		24,70	17,15	1,33
*Stockholm*	427,12	441,22	449,63	456,40	455,75	453,86	458,87	465,14	471,09	479,24	483,04	471,55		350,80	10,40	0,83
*North-Central Sweden*	289,24	295,29	302,33	305,79	322,82	326,97	330,70	335,65	345,05	352,07	355,07	335,94		13,40	16,15	1,26
Switzerland	379,87	384,98	384,74	381,74	383,27	380,72	383,28	391,57	404,00	412,58	419,71	425,06	429,78	212,10	13,14	0,95
*Zürich*			468,36	460,78	464,45	462,64	462,63	472,34	486,92	501,13	510,99	520,78	522,54	904,60	11,57	1,00
*Central Switzerland*			263,37	258,91	262,48	261,35	270,33	274,12	286,75	297,72	304,29	309,23	314,70	188,70	19,49	1,63
*Average*	**314,78**	**321,92**	**324,96**	**321,78**	**324,62**	**328,60**	**334,73**	**341,66**	**347,52**	**352,96**	**355,98**	**361,59**	**370,68**		**17,80**	**1,27**

*Notes*: *PD* population density, *PC* Percentage change; ^a^ break in time series

Work status of physicians: practicing, except for Greece and Portugal with data on physicians licensed to practice; Switzerland (up to 2007), Turkey, Finland (NUTS 2 level) with data on prof. active), France (data from 2011 onwards refer to practicing physicians, data at NUTS 2 level refer to prof. active). Deviation from definition: Belgium (stomatologists included), Croatia (maxilla facial surgeons included), France (interns and residents are not included). Difference in methodology: Finland (estimation based on 2014 survey according to which 91,5% of prof. Active are practicing physicians), Slovakia (physicians at NUTS 2 level are divided from total number of prof. active)

Reference period: data at 31st of December, except for the Netherlands (last Friday before Christmas), Sweden (1st of November)

Source: [30]

### Growth of the physician workforce at national and regional level between 2005 and 2017

Analysis of growth rates at regional level showed that physician rates increased in 117 out of 121 regions. Growth rates were highest in regions with low initial physician supply: in Romania (South-West Oltenia CAGR 2008–2017: 4.04%), Denmark (Sjaelland CAGR 2007–2016: 3.37%), Slovenia (Eastern Slovenia CAGR 2008–2017: 3.25%), Hungary (Central Transdanubia CAGR 2005–2017: 3.21%), Croatia (Adriac Croatia CAGR 2010–2017: 2.65%) and Austria (Burgenland CAGR 2005–2017: 2.22%) (Table 2). Yet, four regions in four different countries have seen a decrease of physician densities: Central Bohemia in the Czech Republic (CAGR 2005–2013: − 1.05%), Flevoland in the Netherlands (CAGR − 0.59%), Wielkopolskie in Poland (CAGR 2005–2016: − 0.91%) and Central Hungary, the region with the capital Budapest (CAGR 2005–2016: − 0.49%).

Despite comparatively higher growth rates in several regions with originally low density levels in six countries (as above), within-country differences of physician ratios have widened between 2005 and 2017 in eight countries (Belgium, Croatia, Czech Republic, Netherlands, Germany, Romania, Poland, Slovakia), while differences decreased in seven countries (Austria, Bulgaria, Denmark, Hungary, Slovenia, Sweden, Switzerland). The widening of within-country disparities in some countries was in part due to the rapid growth of physician ratios in regions with high initial physician supply, namely in metropolitan regions in the Netherlands, Germany and Slovakia (Utrecht, Hambourg, Bratislava) (Table 2). In Romania, within-country differences have widened despite high growth rates in some regions (with low initial physician density) also due to continuously low physician density in South-Muntenia, the surrounding area of the capital region Bucharest-Ilfov. Also, cross-country differences of physician density rates increased in this period. The difference of physician per population rates between Poland, the country with the lowest physician density both in 2005 and 2017, and Austria, the country with the highest density in 2005 and 2017, increased from 217.9 in 2005 to 276.7 per 100,000 population in 2017.

Similar to the trends at regional level, all countries showed a continuous growth of their physician workforce at national level. The average physician density increased from 314.7 practicing physicians per 100,000 population in 2005 to 370.7 in 2017 (or latest available year), which is an increase of CAGR 1.27%. Growth rates were highest in countries that started with comparably low physician densities, namely in Romania (CAGR 2008–2017: 2.83%), the Netherlands (CAGR 2.18%) and in Slovenia (CAGR 2.17%). In contrast, physician rates only increased marginally in the Czech Republic (CAGR 2005–2013: 0.43%) and in Belgium (CAGR 0.60%) (Table 2).

### Density of nurses and midwives

**Fig. 2:**
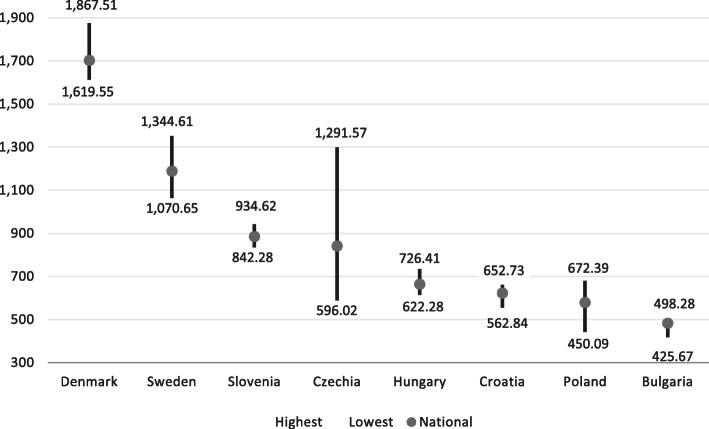
Nurse and midwife distribution by national level and region (NUTS 2) per 100,000 population in eight countries in 2015 (or latest available year). Notes: Data end points refer to the region (NUTS2) with the highest and lowest density of nurses and midwives, and national average across all NUTS2 regions, Year 2015, except for: Denmark (2014) and Sweden (2014). Regions with highest/lowest physician densities by country: Denmark (North Jutland/Capital region), Sweden (Upper Norrland/Stockholm), Slovenia (West Slovenia/East Slovenia), Czechia (Praha/Central Bohemia), Hungary (South Transdanubia/Central Transdanubia), Croatia (Contintental Croatia/Adriatic Croatia), Poland (Lubelskie/Wielkopolskie), Bulgaria (North-West/North-East). Sources: EUROSTAT 2019 data on nurses and midwives by NUTS2 region

Across the eight countries included in the analysis for nurses and midwives, there were on average 866.6 nurses and midwives per 100,000 population in 2014.^1^ The Nordic countries had the highest nurse and midwife rates, about twice as high as the average nurse and midwife density: Denmark showed the highest density with 1702.5 nurses and midwives, followed by Sweden (1188.5). Countries with the lowest national densities were Bulgaria (483) and Poland (578.8). Across all countries, the national differences of nurse and midwife densities were higher than for physicians, varying by a factor of 3.5 between Denmark and Bulgaria (Fig. 2 and Table 3).

**Table 3:** Geographical supply of nurses and midwives (practicing) between highest and lowest density regions and national average, 2005–2015 (or nearest) (per 100,000 population)

Country / *region highest/lowest region*	2005	2006	2007	2008	2009	2010	2011	2012	2013	2014	2015	PD (per sq. km), 2015	PCchange (2005–2015, or nearest)	CAGR (2005–2015 or nearest)
Bulgaria	452,93	460,83	472,41	475,81	473,51	473,70	474,65	483,86	491,82	486,99	483,02	66,2	6,64	0,59
*North-West*	459,25	464,81	481,08	486,64	487,26	494,99	486,10	490,34	496,49	501,01	498,28	42,5	11,00	0,74
*North-East*	420,27	436,75	446,00	452,38	454,31	446,69	442,57	434,92	435,57	426,26	425,67	65,5	1,28	0,12
Croatia	532,83	541,50	553,06	573,76	561,99	581,89	595,04	605,94	620,83	616,99	622,86	74,4	16,90	1,43
*Cont. Croatia*						599,14	615,03	627,26	645,28	641,92	652,73	88,1	8,94	1,44
*Adriatic Croatia*						546,76	554,40	562,67	571,35	566,71	562,84	56,6	2,94	0,48
Czechia	853,51	848,66	844,65	838,45	850,96	851,91	846,10	848,29	841,00	834,01	841,49	136,6	−1,41	−0,13
*Praha*	1.339,12	1.331,16	1.334,91	1.320,76	1.342,13	1.331,29	1.309,12	1.316,97	1.311,98	1.292,98	1.291,57	2604,7	−3,55	−0,33
*Central Bohemia*	641,15	638,19	619,49	610,54	619,28	607,81	602,75	603,85	600,20	593,74	596,02	122,3	−7,04	−0,66
Denmark	1.463,40	1.473,77	1.454,96	1.517,45	1.588,82	1.611,90	1.630,28	1.661,63	1.683,74	1.702,46		132,4	16,34	1,52
*North Jutland*	n/a	n/a	1.548,70	1.657,30	1.756,68	1.818,78	1.862,35	1.878,36	1.863,90	1.867,51		74,1	20,59	2,37
*Capital region*	n/a	n/a	1.412,73	1.450,92	1.528,41	1.533,23	1.549,10	1.587,68	1.614,49	1.619,55		695,1	14,64	1,72
Hungary	614,58	639,32	610,87	631,94	638,35	639,09	638,42	649,75	659,65	658,35	664,09	107,9	8,06	0,71
*South Transdanubia*		714,31	658,79	677,65	715,95	680,62	686,28	784,32	750,11	704,25	726,41	65,8	1,69	0,17
*Central Transdanubia*		529,16	552,46	574,19	580,89	572,25	583,67	590,26	593,78	604,05	622,28	99,4	17,60	1,63
Poland	564,19	564,40	575,30	577,49	584,74	587,19	587,35	620,04	587,33	582,90	578,75	123,6	2,58	0,23
*Lubelskie*	623,58	585,06	603,37	612,42	612,51	654,52	634,49	694,47	675,08	649,70	672,39	85,1	7,83	0,69
*Wielkopolskie*	498,57	495,22	482,16	485,61	457,94	488,07	470,28	521,22	475,14	476,95	450,09	117,0	−9,72	−0,93
Slovenia	750,62	763,43	778,98	792,01	806,99	823,55	838,54	822,01	837,74	862,76	885,71	102,4	18,00	1,52
*West Slovenia*				866,00	878,74	887,84	902,10	875,95	883,30	924,11	934,62	124,3	7,92	0,96
*East Slovenia*				729,14	745,43	768,00	783,36	774,91	797,66	808,50	842,28	88,6	15,52	1,82
Sweden	1.145,25	1.160,76	1.171,57	1.175,26	1.175,75	1.183,61	1.185,64	1.189,41	1.190,93	1.188,49		24,1	3,78	0,37
*Upper Norrland*	1.260,83	1.290,27	1.309,08	1.325,61	1.326,04	1.331,02	1.342,77	1.346,23	1.348,63	1.344,61		3,4	6,64	0,65
*Stockholm*	1.051,07	1.060,96	1.070,27	1.074,09	1.074,13	1.078,08	1.075,35	1.072,42	1.070,15	1.070,65		339,4	1,86	0,18
Average	**797,16**	**806,58**	**807,73**	**822,77**	**835,14**	**844,11**	**849,61**	**860,12**	**864,13**	**866,62**	**679,32**		**8,71**	**0.84**^**4**^

Notes: *PD* population density, *PC* Percentage change, *CAGR* Compound Average Growth Rate

Work status of nurses and midwives: practicing, except for France (nurses), Portugal, Slovakia and Turkey with data on prof. Active. Deviation from definition: Austria (data refer only to nurses and midwives employed in hospitals), Portugal (nurses who hold a post / job under which nursing education is not required are not excluded. Difference in methodology: Czech Republic (double counting for nurses and midwives working in more than one establishment), Netherlands (until 2008 estimates derived from all registered economically active nurses, from 2014 data refer to nurses and midwives who are licensed to practice, requiring that they have been practicing in the past five years). Reference period: Data at 31st of December, except for the Netherlands (from 2014, last Friday before Christmas), Sweden (1st of November), Norway (3rd week of November), Turkey (no reference period given)

Source: [30]

2014 is used for the averge as no data is availabe for Denmark and Sweden for 2015. The average for 2015 is much lower (679.32) given that Denmark and Sweden have the highest per capita ratios of nurses and midwives.

Similar to physicians, there were also large differences of nurse/midwife per population rates across regions, ranging from 1867.5 nurses and midwives per 100,000 population in Denmark (North Jutland) to 425.7 in Bulgaria (North-East) (Fig. 2), equal to a 4.4-fold difference. Within countries, the largest geographical imbalance of nurses and midwives was reported in the Czech Republic (2.2-fold) (see Fig. 2).

In five of the eight countries included, the concentration of nurses and midwives was higher in less populated areas while in more densely populated areas densities were lower. In particular, in the Nordic countries, there was a clear inverse relationship of the urban-rural divide that was observed for physicians. In Sweden and Denmark, regions with lowest population densities showed the highest nurse and midwife rates while capital regions with highest population densities had the lowest rates. For example, the most sparsely populated region in northern Sweden (Upper Norrland with 3.4 people per sq. km) showed the highest nurse/midwife ratio (1344.6) while the capital area with highest populated density (Stockholm 339.4 people per sq. km) had the lowest nurse densities in 2014 (1070.7). Also, regions in Bulgaria (North-West), Hungary (South Transdanubia) and Poland (Lubelskie), among the most sparsely populated regions, had highest nurse and midwife density rates. Only in three countries, the Czech Republic, Croatia and Slovenia the density of nurses was similar to those of physicians, with the highest nurse/midwife rates in capital regions. However, the geographical aggregation in Slovenia and Croatia consisted of only two NUTS 2 regions and does not allow for an in-depth distinction between areas (Table 3).

### Growth of the nurse and midwife workforce at national and regional level between 2005 and 2015

Over time, density rates among nurses and midwives increased in all eight countries from on average 797.2 nurses and midwives per 100,000 population in 2005 to 866.6 in 2014^2^ (CAGR 2005–2014: 0.84%). The relatively low increase is mainly linked to negative growth of nurse and midwife density rates in the Czech Republic (CAGR 2005–2013: − 0.13%) and moderate growth rates in Poland and Sweden. Countries with the strongest increase of nurses and midwives per population are Slovenia (CAGR 2005–2015: 1.52%), Croatia (CAGR 2005–2015: 1.43%) and Denmark (CAGR 2005–2014: 1.52%) (Table 3). Out of the 54 regions included in the analysis, 11 regions reported a negative compound growth rate of nurse and midwives between 2005 and 2015. Five of these regions are in Poland, five in the Czech Republic and one in Hungary. Five of these areas already had among the lowest nurse and midwife density levels in 2005. Similar to physicians, the divergence of national nurse and midwife densities between countries with the highest and lowest densities both in 2005 and 2014 (Bulgaria and Denmark) has become larger, increasing from 1010.5 in 2005 to 1215.5 nurses and midwives per 100,000 population in 2014.

## Discussion

### Density of health professionals within and across countries

This study found large differences in the supply of health professionals across countries in Europe, with differences being especially pronounced at regional level. Among the countries studied, there was a 2.4-fold difference in the density among physicians and a 3.5-fold difference among nurses and midwives between the highest and lowest density countries based on national averages. In contrast, regional density differences across NUTS2 regions were as large as 5.5-fold for physicians and 4.4-fold for nurses and midwives and did not improve over the ten-year time period studied. All capitals and/or major cities showed a markedly higher supply of physicians than sparsely populated regions. However, this was not the case for the density of nurses and midwives, which was mixed, it tended to be higher in less populated areas in five of the eight countries covered.

Our results show that in all countries physician density levels are highest in densely populated regions, such as those with capitals and agglomerations. This is congruent with previous research showing that physicians prefer to practice in affluent urban settings [9, 11] where larger numbers of specialist practitioners are concentrated. These regions generally have higher attractiveness in regard to transport infrastructure, careers for spouses, leisure opportunities, better access and availability of education and childcare facilities, and employment opportunities with higher prestige [11, 36–39]. For instance, in the Czech Republic and Romania, a large number of physicians are attracted to practicing in the densely populated of the capital cities of Prague and Bucharest. Prague has the highest staffing of physicians, nurse and midwifes while Central Bohemia, a surrounding area of Prague, has the lowest density levels. This also holds to the capital region of Bucharest, Bucharest-Ilfov, and the surrounding region of South Muntenia. Both countries have metropolitan systems where the cities are the capital and the largest urban centre at the same time, creating a high degree of polarization and dominance including attraction of highly skilled human capital from surrounding areas [40–42].

The trend of higher density levels in urban areas compared to rural areas does not consistently hold for nurses and midwives. Our analysis of the eight countries showed that nurses and midwives are particularly concentrated in regions with low population density except for three countries (Czech Republic, Croatia and Slovenia). A number of underlying reasons might explain this relationship. First, rural areas tend to have an older population which leads to more demand for nursing care while physician density tends to be lower in areas with an older population structure. For example, one study found that general practicioner (GP) density was positively associated with the share of the population aged 60 and above within metropolitan areas, but negatively within rural areas in Germany [43]. Second, the education of physicians in many countries tends to be located in larger cities with university hospitals. Yet, the education of nurses and midwives is often more decentralized and takes place in cities as well as in regions. For example, in Sweden 21 public sector universities or university colleges and four independent education providers offering education for registered nurses [44], while there are only seven universities authorized to educate physicians [45]. Third, working and living conditions can be highly influencing factors for the decision of nurses and midwives of their place of work [46]. Working in hospitals in some large cities show to be less attractive for nurses due to high and rising costs of living and the lack of affordable housing given their remuneration [47, 48]. Some nurses in urban areas may also choose alternative (non-nursing) employment with better renumeration, career development and working environment [46, 47]. Fourth, the inverse relationship of physician and nurse/midwife densities may relate to how countries allocate roles, tasks and responsibilities between physicians and nurses/midwives. In order to alleviate primary care provider shortages, many European countries have implemented task shifting from physicians to nurses with additional training, such as advanced practice nurses (APNs). APNs are authorized to perform an expanded set of clinical activities [49, 50].

### Geographical distribution over time

Results across time showed an increase in the density levels of physicians, nurses and midwives in all countries between 2005 and 2017 or latest years available. However, physician rates grew faster than rates of nurses and midwives. This is linked to the rapid increase of physician density rates in urban areas with already high-density levels and the modest growth of physician rates in some regions with low initial physician density. Density rates among nurses and midwives grew comparatively slower, due to the higher number of regions with negative growth of nurse and midwife density levels. Negative growth rates among nurses and midwives, particularly observed in the Czech Republic and Poland, have been found to be multi-factorial. Influencing factors include ineffective planning, low salary levels, high workloads, migration of nurses and the freeze in recruitment of health professionals in the public sector following the 2008 economic crisis [39, 51, 52].

The described trends of health professionals-to-population ratios over the last decade might also, in part, reflect country differences in recruitment and retention planning [14]. For example, in Austria, no systematic health workforce planning mechanism exists, except the admission criteria for first year students to public medical universities. The supply of physicians has been left to market developments, with the result that Austria has among the highest physician densities across the EU with disparities at regional level, in particular among specialists [53].

Regional imbalances in the supply of healthcare professionals can affect adequate access to health services and lead to disparties in health. For example, Rosenthal et al. [54] demonstrated that residents of metropolitan areas have better geographic access to physicians. A recent study on subjective perceptions of unmet need for health care in Europe showed that low physician density was associated with unmet need due to availability [55]. Several studies demonstrated the association between low nurse staffing levels, negative health outcomes and lower patient satisfaction [56–58]. Nurse shortages have been recognized as a pressing policy issue resulting from inadequate workforce planning, allocation decisions and austerity measures [46, 57].

Future research should pay greater attention to regional workforce imbalances and the impact that policies and programmes can have on improving worker distributions, both within countries and internationally [11, 59].

## Limitations

This study has several limitations. First, NUTS 2 regions can cover large territories which may comprise densely and sparsely populated areas (e.g. Germany, Spain and the Netherlands) and data at NUTS 3 level were not available. The limitation of additional disaggregation of data to lower geographical units has led to few regions being entirely or primarily rural, whereas the capital cities were usually classified as urban. Hence, the results probably underestimate supply levels for some rural regions. Similarly, the results in small countries with few NUTS 2 regions are limited.

Second, only eight countries could be covered in the analysis for nurses and midwives, due to data availability, limiting the generalizability of the findings. Moreover, the aggregation of nurses and midwives into a single indicator in EUROSTAT’s regional statistics does not allow separate analyses for these two professions.

Third, our analysis was restricted to data available in the EUROSTAT database, resulting in a limited number of comparable countries, in particular for nurses and midwives. Strengthening data collection at regional level and ensuring improved data comparability would allow for a better identification of the geographical distribution in Europe. Despite these limitations, the study provides valuable insights into the stark variations in regional distributions of physicians and nurses and midwives across a selection of European countries.

## Conclusions

This study found markedly higher regional imbalances than cross-country differences in density levels for both physicians, and nurses and midwives. Time trends over a ten-year period showed no improvements in regional distributions over time. Physicians practiced more frequently in urban areas. Nurses and midwives practiced in less densily populated areas at similar (and at times higher) rates, than more polulated areas, but based on only eight countries covered. Future research is needed to analyse geographical imbalances in more countries and examine potential explanations for these patterns, including demographic characteristics of health care workers, working and living conditions, availability and distribution of educational institutions, and variations in scope of practice among health care professions. Moreover, policy makers and health workforce planners should take into account the impact of allocation decisions on staffing levels across regions and consequences for quality of care. Appropriate data on both regional supply and employment of health professionals are key for these policy decisions and integrated workforce planning.

Agencies involved with the collection of international health workforce data should improve the data collection at sub-regional levels, ideally at NUTS 3 level, to improve the supply estimation of the health workforce including nurses, midwives and allied health professionals across different population densities. Better data would enable workforce planners and policy makers to make more appropriate workforce planning taking into account regional variations in supply [59]. Integrated workforce planning at national and regional levels, linked to workforce policies and programmes, would allow to meet the needs of populations locally and nationally to ensure timely access to quality care for all.

## Data Availability

The data on the numbers of physicians, nurses and midwives per population from EUROSTAT are publicly available. The datasets analysed during the current study are availabe in EUROSTAT under: Health personnel (medical doctors & nurses and midwives) by NUTS 2 regions, Per 100 000 inhabitants: https://appsso.eurostat.ec.europa.eu/nui/show.do?dataset=hlth_rs_prsrg&lang=en

## Abbrevations

APN: Advanced Practice Nurse
CAGR: Compound Annual Growth Rate
EU: European Union
EFTA: European Free Trade Association
GP: General practicioner
HP: OECD/EUROSTAT classification of Health Care Providers
LFS: Labour Force Survey
LP: (physicians, nurses, midwives) licensed to practice
NUTS: Nomenclature of Territorial Units for Statistic
P: Practicing physicians, nurses, midwives
PA: Professionally active physicians, nurses, midwives
PC: Percentage change
PD: Population density

## Footnotes

1 2014 is used for the averge as no data is availabe for Denmark and Sweden for 2015. The average for 2015 is much lower (679.32) given that Denmark and Sweden have the highest per capita ratios of nurses and midwives.

2 2014 is used for the averge as no data is availabe for Denmark and Sweden for 2015. The average for 2015 is much lower (679.3) given that Denmark and Sweden have the highest per capita ratios of nurses and midwives.
